# An elderly case of paraneoplastic anti-NMDA receptor encephalitis associated with large cell neuroendocrine carcinoma of the lung

**DOI:** 10.1186/s12890-024-03137-z

**Published:** 2024-07-12

**Authors:** Masamitsu Naka, Atsushi Inaba, Hana Miyasaka, Keisuke Suzue, Junichi Ishigaki, Hideki Shibuya, Kei Hara, Nobuya Ohishi, Yusuke Sugiyama, Yasushi Shiio, Ryosuke Tajiri, Yukiko Kishida, Tasuku Ishihara, Akihiro Yugeta

**Affiliations:** 1 Department of Respiratory Medicine, Tokyo Teishin Hospital, 2-14-23 Fujimi, Chiyoda-ku, Tokyo, 102-8798 Japan; 2 Department of Neurology, Tokyo Teishin Hospital, 2-14-23 Fujimi, Chiyoda-ku, Tokyo, 102-8798 Japan; 3 Department of Pathology, Tokyo Teishin Hospital, 2-14-23 Fujimi, Chiyoda-ku, Tokyo, 102-8798 Japan; 4 Department of Neurology, National Center of Neurology and Psychiatry, 4-1-1 Ogawa-Higashi, Kodaira, Tokyo, 187-8551 Japan

**Keywords:** Paraneoplastic anti-NMDA receptor encephalitis, Large cell neuroendocrine carcinoma, Immunohistochemistry

## Abstract

**Background:**

Recent studies have suggested that N-methyl-D-aspartate (NMDA) receptors are involved in the cell proliferation in several tumors. However, there have been no reports demonstrating the expression of NR1 subunit of the NMDA receptor in large cell neuroendocrine carcinoma (LCNEC).

**Case presentation:**

Here, we report the first elderly case of paraneoplastic anti-NMDA receptor encephalitis associated with LCNEC of the lung with NR1 expression. Of note, NR1 subunit expression in the tumor cells of the present case was confirmed by immunohistochemistry (IHC). Radiation therapy and immunotherapies, such as corticosteroids and intravenous immunoglobulin (IVIG), shrank the tumors and improved neurological symptoms in the present case. Additionally, we also confirmed the expression of NR1 in the tumor cells obtained from three other cases with LCNEC of the lung at our hospital by IHC.

**Conclusion:**

Our IHC results indicate that LCNEC generally expresses NR1 subunit and NMDA receptor may be involved in the tumor development and growth.

## Background

N-methyl-D-aspartate (NMDA) receptor is one of the ionotropic glutamate receptors, which mainly expresses in the central nervous system (CNS) and plays roles in synaptic plasticity, memory and learning. [1] Recently, NMDA receptor has been found in a variety of peripheral tumor tissues outside the CNS, such as melanoma, prostate cancer, colon cancer, thyroid cancer, gastric cancer, esophageal cancer, and small cell lung cancer (SCLC) [2–4]. The study using melanoma cells showed that NMDA receptors are present in the nuclei as well as in the cytosols of the melanoma cells. [2] The NMDA receptor activation is supposed to contribute to the development of human malignancies, through some cell-proliferation signaling activities such as mTOR pathway that controls cell growth. [2, 5, 6] Therefore, NMDA receptor is an attractive target for anti-cancer drug development, like other glutamate receptors. [5]

Anti-NMDA receptor encephalitis is an autoimmune disorder induced by IgG antibodies against the NR1 subunit of the NMDA receptor. [7] Anti-NMDA receptor encephalitis develops in patients of all ages associated with the tumors with neuroendocrine differentiation, but more frequently in young adults and children with teratoma. [7] Large cell neuroendocrine carcinoma (LCNEC) is a rare subgroup of high-grade neuroendocrine cancer that can occur throughout the body. Previously, LCNEC arising from the uterine corpus was reported to be one of the causes of anti-NMDA receptor encephalitis. [8] We herein report the first elderly case of paraneoplastic anti-NMDA receptor encephalitis associated with LCNEC of the lung with NR1 expression. Additionally, NR1 expression was also confirmed in the tumor cells obtained from three other cases with LCNEC of the lung at our hospital. It is noteworthy that this is the first report to show the expression of NR1 subunits in the tumor cells of LCNEC by immunohistochemistry (IHC).

## Case presentation

**Fig. 1:**
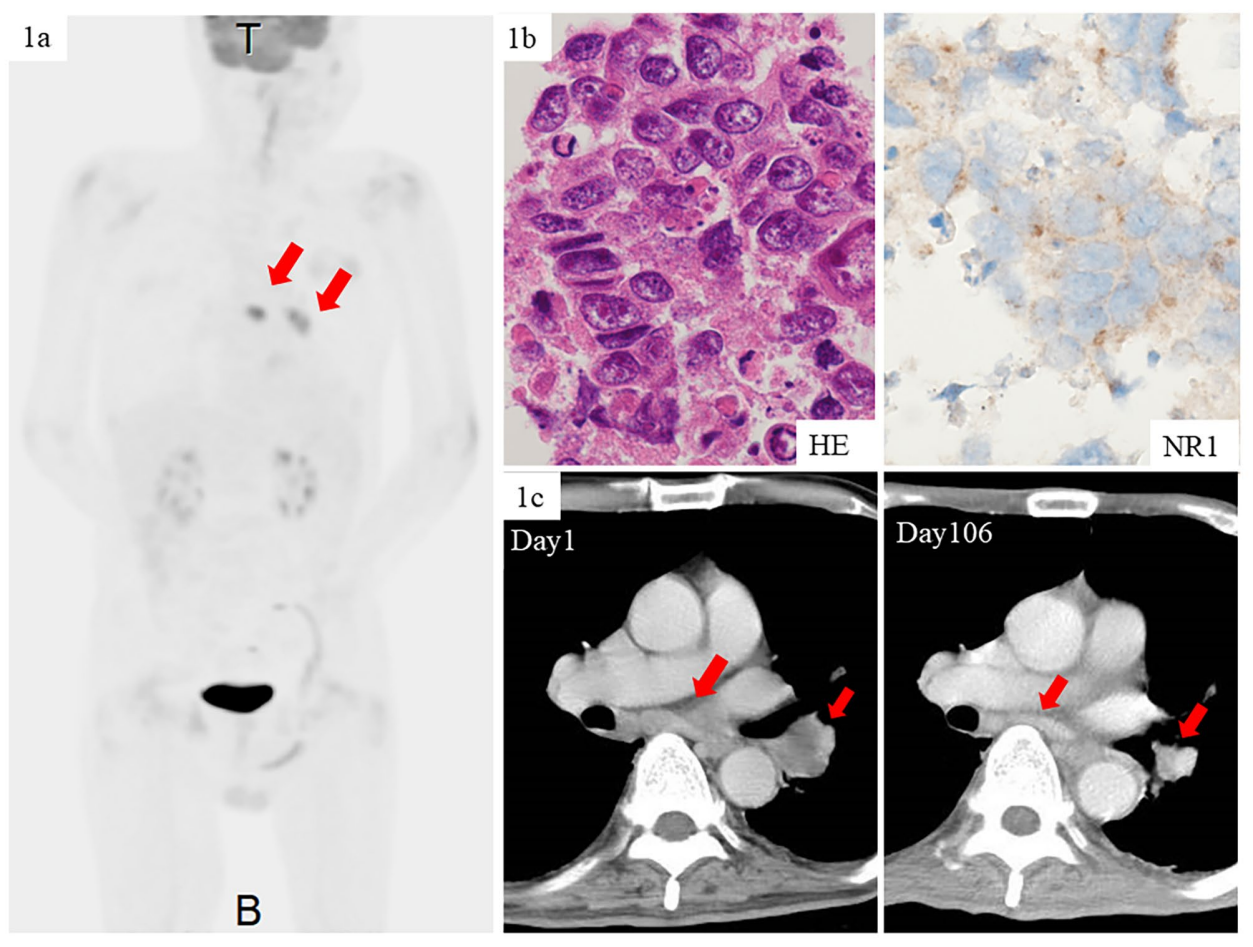
(**1a**) ^18^F-FDG-PET/CT showed high FDG uptake in the left hilar mass with an enlarged mediastinal lymph node. (**1b**) Hematoxylin and Eosin staining revealed that large-sized neoplastic cells with distinct cytoplasm proliferated diffusely. NR1 subunit expression in the tumor cells was confirmed by IHC. (**c**) The tumors shrank after radiation therapy (39 Gy/13 fr) and IVIG (400 mg/kg/day)

A 72-year-old Japanese man with a history of 50-pack-year smoking visited a local hospital with abnormal behaviors such as wandering, depression, deficits in short-term memory and partial seizure. No abnormalities were found in magnetic resonance imaging (MRI) of the brain, computed tomography (CT) of the trunk and blood tests. Electroencephalogram (EEG) revealed no findings indicating epilepsy. Ten days after consultation, he was admitted to the hospital, due to worsening consciousness disturbance　(Glasgow Coma Scale E1V1-2M1). He didn’t have a fever, signs of meningeal irritation and inflammatory reactions in blood tests. The cerebrospinal fluid (CSF) contained 12 white blood cells/mm^3^ without malignant cells, protein 72 mg/dL (normal range 10-50 mg/dL) and IgG index 1.15 (normal range < 0.7) and normal level of glucose 67 mg/dL (blood sugar 103 mg/dL). Therefore, he was diagnosed with some type of immune-mediated encephalitis and treated with one cycle of high-dose intravenous methylprednisolone (1 g/day for 3 days) and levetiracetam. However, his neurological symptoms did not improve. He was transferred to a neurological hospital for further examination. MRI of the brain didn’t show any abnormal signs. Brain metastases were ruled out by contrast-enhanced MRI. ^18^F-fluorodeoxyglucose (^18^F-FDG)-positron emission tomography (PET)/computed tomography (CT) showed high FDG uptake in the left hilar mass with an enlarged mediastinal lymph node (Fig. 1a). EEG detected diffuse slow waves. Autoimmune encephalitis associated with lung cancer was suspected, thus he was transferred to our hospital for further evaluation and treatment.

On admission, his temperature was 37.7℃, blood pressure 128/81 mmHg, pulse 72 beats per minute, and oxygen saturation 96% while he was breathing ambient air. On neurological examination, he was unresponsive with eyes open (Glasgow Coma Scale E4V1M4). He presented with diminished cough and snout reflex, orofacial and right arm myoclonus, and rigidity in the bilateral arms. Blood tests revealed elevation of serum C-reactive protein (1.60 mg/dL, normal range < 0.3 mg/dL) and progastrin-releasing peptide (ProGRP) (127 pg/mL, normal range < 81.0pg/mL). Tests for serum antibodies (AMPH, CV2, PNA2, Ri, Yo, Hu, Recoverin, SOX1, titin, zic4, GAD65, Tr/DNER, LGL1, GASPR2) were all negative. The CSF contained 7 white blood cells/mm^3^ without malignant cells, glucose 79 mg/dL, albumin 25.2 mg/dL, immunoglobulin G (IgG) 5.1 mg/dL. Elevated IgG index (4.20, normal range < 0.7) was shown and positive oligoclonal band was detected in the CSF. Anti-NR1 subunit antibody was detected in his CSF by cell-based assay. Trans-esophageal ultrasound-guided fine needle aspiration for the enlarged mediastinal lymph node was performed. Microscopic findings of the tumor cells showed that large-sized neoplastic cells with distinct cytoplasm proliferated diffusely (Fig. 1b, left panel), and they were positive for chromogranin A, synaptophysin, and CD56, suggesting neuroendocrine differentiation. Taken together, a metastasis of LCNEC to the lymph node was pathologically confirmed. Furthermore, NR1 subunit expression was confirmed by IHC (Fig. 1b, right panel). Therefore, he was diagnosed with paraneoplastic anti-NMDA receptor encephalitis associated with LCNEC of the lung. The tumor was classified as clinical T2aN3M0 Stage IIIB according to the TNM classification of the Union of International Cancer Control (UICC), 8th edition.

Although treatment was initiated with ampicillin/sulbactam for a tentative diagnosis of aspiration pneumonia, his fevers did not resolve, with elevated levels of CRP. He was suspected of having neoplastic fever and then treated with intravenous betamethasone and prednisolone. After the pathological diagnosis was made, intravenous immunoglobulin (IVIG) (400 mg/kg/day for 5 days) was administered and radiation therapy for the left hilar tumor and mediastinal lymph nodes (39 Gy/13 fr) was performed. Although low blood pressure upon sitting position and orofacial myoclonus remained, his disturbed consciousness and arm myoclonus gradually improved. He started following commands and responding partially in a conversational manner (Glasgow Coma Scale E4V5M6). In parallel with these symptomatic improvements, the tumors shrank (Fig. 1c), and the ProGRP level was decreased.

## Clinicopathological characteristics of patients

Tumor tissues were obtained from four patients including the present case, who had been clinically and pathologically diagnosed with LCNEC of the lung at our hospital from 2006 to 2021. The clinicopathological characteristics of the four patients were presented in Table 1. All patients were male in their 70s with heavy smoking history. The present case (Case 1) was the only patient with neurological symptoms.

**Table 1:** Clinical characteristics of the four patients with LCNEC of the lung

Case	1	2	3	4
Sex	male	male	male	male
Age	72	70	78	78
smoking history (pack-year)	50	70	30	45
neurological symptoms	abnormal bevavior, speech dysfunction, disturbance of consciousness, seizures, rigidity postures, autonomic dysfunction	none	none	none
biopsy tissues	lymph node metastasis	hepatic metastasis	hepatic metastasis	primary lesion
Immunohistochemical examination chromogranin A	positive	positive	positive	positive
Immunohistochemical examination synaptophysin	positive	positive	positive	positive

**Fig. 2:**
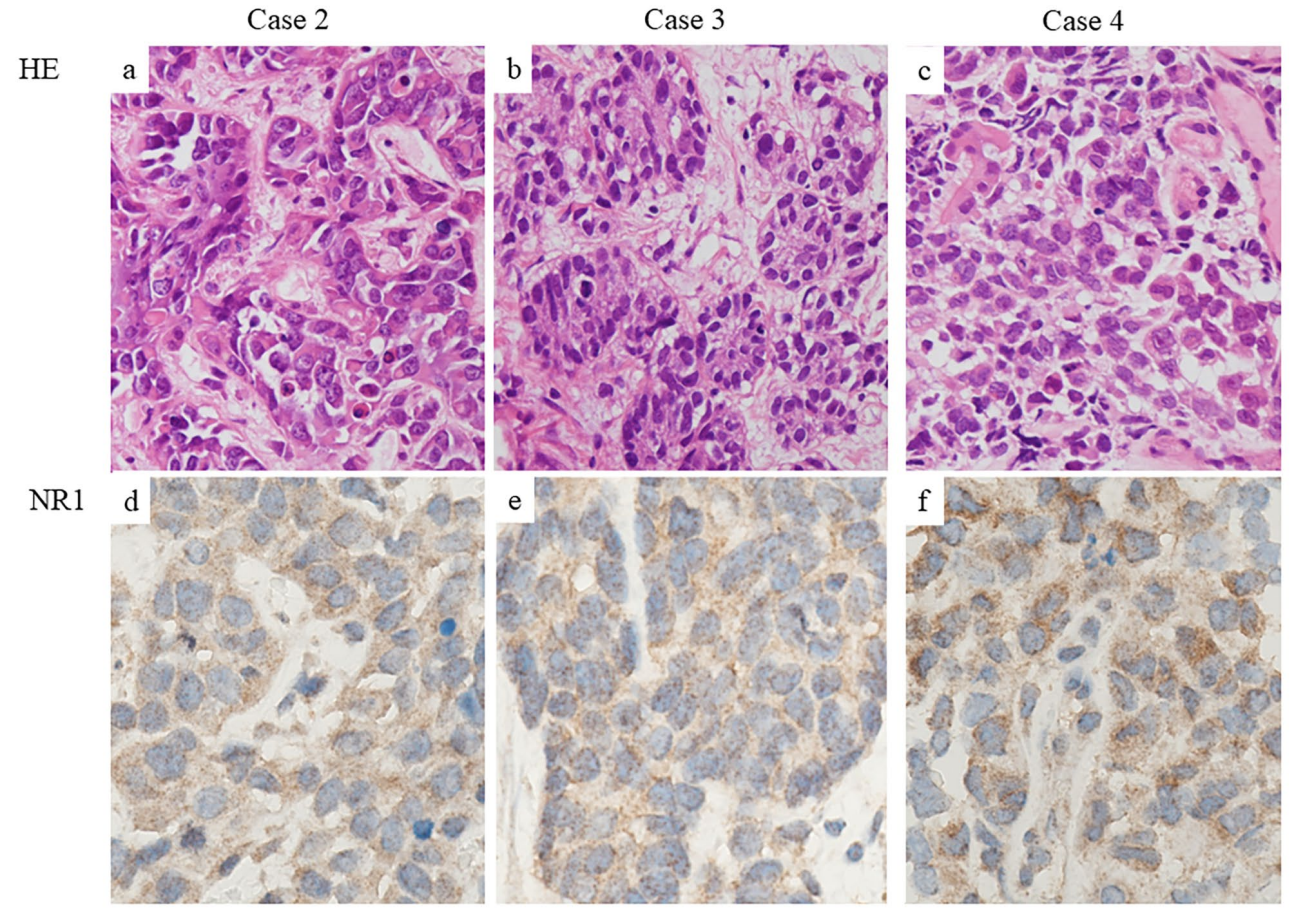
(**a**, **b**, **c**) Hematoxylin and Eosin staining revealed nests of large-sized neoplastic cells with ample cytoplasm and distinct nucleoli. (**d**, **e**, **f**) IHC for NR1 subunits showed paranuclear and nuclear dot-like staining (Case 2, a&d; Case 3, b&e; Case 4, c&f;)

Case 2 was diagnosed by needle biopsy of the hepatic metastases. Radiological examination revealed a tumor in the right hilum of the lung, with metastases to bilateral adrenal glands and the liver. The tumor was classified as clinical T2N2M1 Stage IV according to the TNM classification of UICC, 6th edition. Although he received 3 courses of carboplatin (CBDCA) and paclitaxel and 1 course of cisplatin and docetaxel, he had progressive disease (PD). He died 4 months after diagnosis. Case 3 was also diagnosed by needle biopsy of the liver metastases. Radiological examination revealed a primary tumor in the left upper lobe, with metastases to the lung, brain, liver, and bone. The tumor was classified as clinical T4N0M1b Stage IV according to the TNM classification of UICC, 7th edition. In spite of receiving 3 courses of CBDCA and etoposide, he also developed PD. He died 3 months after diagnosis. Case 4 was diagnosed by a transbronchial lung biopsy of the primary lesion. Radiological examination revealed a lesion only in the left lower lobe, and no metastasis was detected. The tumor was classified as clinical T1cN0M0 Stage IA3 according to the TNM classification of UICC, 8th edition. He underwent video-assisted thoracoscopic lobectomy of the left lower lobe and lung lymph node dissection. However, 11 months after surgery, ^18^F-FDG-PET/CT showed FDG uptake in a metastatic pulmonary nodule in right upper lobe, mediastinal lymph nodes, right adrenal gland, pancreas, sigmoid colon, small intestine and mesenteric lymph nodes. He developed small intestinal ileus and acute obstructive cholangitis and died 12 months after diagnosis. Histopathological images of LCNEC (case 2 to 4) are shown in Fig. 2.

## Immunohistochemistry

Antibodies against NR1 (clone 54.1, Invitrogen, ThermoFisher Scientific, Massachusetts, USA; working dilution of 1:50), chromogranin A (clone LK2H10, Ventana medical systems, Arizona, USA; ready-to-use), synaptophysin (clone 27G12, Leica Biosystems, Newcastle, UK; working dilution of 1:100) were used. IHC was done on the Benchmark Ultra system (Roche Diagnostics, Indiana, USA). Both chromogranin A and synaptophysin were positive in all LCNECs. IHC for NR1 showed nuclear dot-like staining as well as cytoplasmic staining in all cases (Fig. 2d to f).

## Discussion

We present the first case of anti-NMDA receptor encephalitis associated with LCNEC of the lung. Anti-NMDA receptor encephalitis has rarely been reported in patients with lung cancer. In a large cohort study involving 577 patients with anti-NMDA receptor encephalitis, 220 had an underlying neoplasm (38%) and 207 tumors were ovarian teratomas (94%).^9^ SCLC is the most common form of lung cancer presenting with paraneoplastic neurological syndrome. A previous study reported that 24 of 264 patients (9.1%) with SCLC presented with paraneoplastic neurological syndrome. [9] However, anti-NMDA receptor encephalitis associated with SCLC has rarely been reported. [10] Although LCNEC in the uterus and pancreas has been reported to present with anti-NMDA receptor encephalitis [8, 11], to the best of our knowledge, no pulmonary LCNEC associated anti-NMDA receptor encephalitis has been reported.

Radiation therapy and immunotherapy with a combination of IVIG and high dose corticosteroids shrank his tumors and improved neurological symptoms. The current hypothesis on the etiology of paraneoplastic encephalitis including anti-NMDA receptor encephalitis is that the antigens expressed on the tumor cells might cross-react with similar antigens expressed on the cells in the CNS. Therefore, the goal of therapy is to remove tumor cells expressing antigens and the cross-reactive antibodies. On the basis of the hypothesis described above and a previous case series study, [7] a standard treatment for anti-NMDA receptor encephalitis was proposed including various immunotherapies and tumor removal. [12] Corticosteroids plus IVIG or plasmapheresis are the first-line immunotherapy agents, while rituximab and cyclophosphamide either alone or in combination are the second-line therapy. [13] In one case series, patients whose tumor was diagnosed and removed within 4 months of neurological symptom development had better outcomes than the others. [7] The present case also supported the suggestion that even a slight reduction in the tumor volume could ameliorate neurological symptoms in patients with anti-NMDA receptor encephalitis.

Our IHC examination revealed that NR1 subunits are also expressed in LCNEC of the lung regardless of neurological symptoms and showed dotted staining in the nuclei as well as in the cytoplasm of the tumor cells. It was thought that the tumor specific immune response for NMDA receptors in LCNEC might cause the encephalitis. NMDA receptor is expressed in SCLC cells and is involved in the tumor development and growth. [3] In addition, it is present in the nuclei of the melanoma cells and may be involved in malignant transformation. [2] Taken together with these observations, our IHC results of NR1 in LCNEC of the lung indicates that NMDA receptor is generally expressed in LCNEC and may be involved in the tumor development and growth.

## Conclusions

We report the first case of paraneoplastic anti-NMDA receptor encephalitis associated with LCNEC of the lung. IHC results showed that LCNEC generally expresses NR1 subunits, suggesting that NMDA receptor may be involved in its malignancy.

## Data Availability

The data and materials are available from the corresponding author on reasonable request.

## Abbreviations

^18^F-FDG-PET: ^18^F-fluorodeoxyglucose-positron emission tomography
CNS: central nervous system
CSF: cerebrospinal fluid
CT: computed tomography
IHC: immunohistochemistry
IVIG: immunoglobulin
LCNEC: large cell neuroendocrine carcinoma
MRI: Magnetic resonance imaging
NMDA receptors: N-methyl-D-aspartate receptors
SCLC: small cell lung cancer
